# Cognition Meets Gait: Where and How Mind and Body Weave Each Other in a Computational Psychometrics Approach in Aging

**DOI:** 10.3389/fnagi.2022.909029

**Published:** 2022-07-08

**Authors:** Francesca Bruni, Francesca Borghesi, Valentina Mancuso, Giuseppe Riva, Marco Stramba-Badiale, Elisa Pedroli, Pietro Cipresso

**Affiliations:** ^1^Faculty of Psychology, eCampus University, Novedrate, Italy; ^2^Applied Technology for Neuropsychology Lab, IRCCS Istituto Auxologico Italiano, Milan, Italy; ^3^Human Technology Lab, Catholic University of the Sacred Heart, Milan, Italy; ^4^Department of Geriatrics and Cardiovascular Medicine, IRCCS Istituto Auxologico Italiano, Milan, Italy; ^5^Department of Psychology, University of Turin, Turin, Italy

**Keywords:** virtual reality, machine learning, aging, artificial intelligence, psychometric, rehabilitation, dual-task, neurology

## Abstract

Aging may be associated with conditions characterized by motor and cognitive alterations, which could have a detrimental impact on daily life. Although motors and cognitive aspects have always been treated as separate entities, recent literature highlights their relationship, stressing a strong association between locomotion and executive functions. Thus, designing interventions targeting the risks deriving from both components’ impairments is crucial: the dual-task represents a starting point. Although its role in targeting and decreasing difficulties in aging is well known, most interventions are focused on a single domain, proposing a vertical model in which patients emerge only for a single aspect per time during assessment and rehabilitation. In this perspective, we propose a view of the individual as a whole between mind and body, suggesting a multicomponent and multidomain approach that could integrate different domains at the same time retracing lifelike situations. Virtual Reality, thanks to the possibility to develop daily environments with engaging challenges for patients, as well as to manage different devices to collect multiple data, provides the optimal scenario in which the integration could occur. Artificial Intelligence, otherwise, offers the best methodologies to integrate a great amount of various data to create a predictive model and identify appropriate and individualized interventions. Based on these assumptions the present perspective aims to propose the development of a new approach to an integrated, multimethod, multidimensional training in order to enhance cognition and physical aspects based on behavioral data, incorporating consolidated technologies in an innovative approach to neurology.

## Introduction

Population aging is one of the most significant difficulties that social and health systems are facing today. According to the World Health Organization, in 2020 the population older than 60 years old represented 13.5% of the world’s population. This number is 2.5 times higher than it was in 1980, and it is growing at an alarming rate (World Health Organization, 2020). The current situation emphasizes the significance of reacting to the constant rise in aging demand by proposing new solutions to challenges arising from the physiological condition to better meet their needs. A significant proportion of older people may develop frailty, multi-morbidity, and disability causing a significant impact on daily life (Lutz et al., 2008; World Health Organization, 2020). The two most frequent conditions of vulnerability in aging are frailty and cognitive impairment: they are described as a pattern of decline in different aspects of motor (i.e., gait, mobility, balance) and cognitive functions (i.e., attention and working memory) (Gobbens et al., 2010), that could increase disability, mortality, reduce the quality of life, and contribute to adverse outcomes (Speechley and Tinetti, 1991; Fried et al., 2004; Rockwood, 2005; Panza et al., 2015; Kojima et al., 2018; de Morais Fabrício et al., 2020) with a direct impact on health as well as on health care and social costs (World Health Organization, 2020). As a result, the strong links between aging and altered motor and cognitive processes should be highlighted, considering also the well-known link between motor and cognitive functions. An example of this relationship is locomotion. Although locomotion appears to be a mechanical effort, it cannot be reduced to a simple action involving a sequence of repetitive movements. This action requires the coordination of gait and one or more cognitive processes at the same time (Yogev et al., 2008; Al-Yahya et al., 2011). In everyday activities, it may be essential to adjust our gait to overcome environmental impediments or just participate in a discussion; these are all requests that necessitate the ability to accomplish many tasks at the same time. According to the Cognitive-Motor Interference theory (CMI) (Montero-Odasso and Speechley, 2018), performing tasks simultaneously, the so-called dual-task (DT), requires a high level of cognitive control in terms of executive processes and attentional abilities whose impairment may produce a deterioration in either motor and cognitive execution or even both (Yogev et al., 2008). As a result, it appears clear how executive functions are an important cognitive resource for normal locomotion. Executive functions are integrative processes involving cognitive and behavioral components that are required for targeted and successful actions, and attentional resource control which are the foundation of the ability to manage autonomous daily activities (Yogev et al., 2008).

Based on these premises, the need to consider both cognition and gait as intertwined aspects is crucial, as said by Giovenale *mens sana in corpore sano*, considering the individual as a whole between mind and body. Despite this consideration has been well known for centuries and the integration of different domains being daily required, in clinical practice motor and cognitive processes often continue to be treated as separate entities, and the intervention methodologies available are frequently provided vertically, focusing on a single aspect at a time. This is a disadvantage and could be attributed to the limitations in considering deficits in aging, which are confined to be assessed and consequently treated separately concerning motor and cognition as in the case of frailty, that most authors operationalized by focusing predominantly on the physical elements (de Vries et al., 2011). Nowadays, researchers are trying to solve the question relative how a healthy body promotes a healthy mind, hence how a healthful mind could influence a good physical state. In this field, a possible solution appears the so-called DT paradigm (Dorfman et al., 2014; Lauenroth et al., 2016). The DT paradigm involves two exercises performed at the same time, particularly a motor task and a cognitive one (e.g., walking while counting backward). Evidence suggests that DT is a powerful tool during both assessment and rehabilitation processes. On one hand, it is more sensitive than a single task to detect some early gait dysfunctions (Lunardini et al., 2021). On the other hand, it benefits a variety of individuals, including older persons with frailty syndrome, neurological disease, and poststroke patients (Deutsch et al., 2013; Wajda et al., 2017; Freitag et al., 2019). However, the most available studies focus on its benefits only for a healthy body—namely, its implications on physical processes. For instance, substantial differences have been shown when comparing CMI with single-task exercise or no intervention on physical outcomes (Wang et al., 2015), such as gait speed, stride length, fall rates, and reaction time. Otherwise, the DT tools used in the neuropsychological field involve the execution of two concurrent tasks engaging only cognitive aspects. An example is to judge the accuracy of alphanumeric equations and practice a simultaneous visual detection task under focused attention (Bier et al., 2018), as well as a single trial usually differs from a DT merely in the presentation of one or two similar stimuli (e.g., discriminating animals and/or celestial bodies by pressing a left or right button, respectively) (Lussier et al., 2012, 2021). Additional problems in the DT paradigm concern the choice of cognitive exercises. During traditional clinical performance the number of tasks that can be completed while performing a motor exercise, like walking, is restricted, especially given the small amount of time it takes to walk a few meters. Recall of words, serial subtraction, and auditory Stroop tests are common examples of activities employed (Al-Yahya et al., 2011; Raffegeau et al., 2019), although walking in a real context requires a great amount of resources such as visual attention, searching strategies, and processing of what is observed. Consequently, operating in a lifelike way is important, considering body and mind as intertwined aspects that could reflect the individual as a whole between mind and body.

## Where Could This Integration Take Place?

Both neuropsychological assessment and training are usually provided in a paper-and-pencil format and they are administered in an isolated and non-ecological setting (Gates and Valenzuela, 2010). They consist of specific domain exercises, such as categorization, classification, etc. addressing a specific ability (memory for proper names, object location, etc.) (Chaytor and Schmitter-Edgecombe, 2003; Wenisch et al., 2007; Gates and Valenzuela, 2010). Computer-based programs are progressively replacing traditional exercises since they allow for multi-modal and multi-domain training, which appears to be a key predictor of functional efficacy (Gates and Valenzuela, 2010; Hu et al., 2021). They also permit personalized intervention in a controlled way by modulating the difficulty level on the individual’s baseline, gradually increasing the difficulty, and tracking results tailored to the patient’s needs. Moreover, computer-based interventions enable the real-time monitoring of cognitive performance as well as the standardization of interventions. The use of commercial packages to improve cognition or physical abilities is extremely prevalent, the Nintendo Brain training and the Brain Age software are some examples (Nouchi et al., 2012; Clemenson and Stark, 2015; Hu et al., 2021), as well as games from the Wii Fit software package (e.g., yoga, soccer, ski jump, tennis) (Franco et al., 2012; Rendon et al., 2012; Bieryla and Dold, 2013).

A further promising step could be offered by novel techniques based on immersive simulations of daily settings, i.e., Virtual Reality (VR). This is a technology that uses 3D computer-generated environments to replicate lifelike situations in an ecological, safe, and controlled situation. The fact that users behave in Virtual Environments (VE) in the same way they would in the real world distinguishes VR from other types of media (Slater and Sanchez-Vives, 2016). This illusion depends on some crucial features such as the feeling of immersion (i.e., the number of senses stimulated, valid actions that are possible within the system, and the reality’s similarity of the stimuli) (Slater, 2009; Cipresso et al., 2018), the sense of presence within the environment (i.e., the feeling of “being there” inside the virtual scenes and tunes its activity accordingly within it) (Riva and Mantovani, 2014; Cipresso et al., 2018; Riva, 2018), and the interaction with objects, which allow to experience ourself as active agents (Sundar et al., 2010; Cipresso et al., 2018; Kim et al., 2019). VR also guarantees a realistic experience through multisensorial displays (e.g., visual, auditory, kinesthetic) and tracking devices that detect any movement and deliver the recorded data to the visualization system. This information about the individual is useful for a real-time update of the VE. Three categories of VR could be identified, based on the degree of immersion and interaction with the VE: non-immersive, semi-immersive, and fully immersive systems (Cipresso et al., 2018; Tuena et al., 2020). The most immersive system enables high active interaction and immersion using a head-mounted display or the Cave Automatic Virtual Environment (CAVE). They can provide a high sense of presence also by isolating individuals, facilitating natural interactions and exchanges that are similar to those found in everyday life (Riva and Mantovani, 2014; Riva, 2018). VR also provides further advantages in terms of integration since it offers the possibility to handle different devices to perform multiple tasks and collect various data. For instance, it is not only possible to cycle or walk with a dedicated bicycle or treadmill while the participant performs a second task in the VE, but also to collect behavioral data during activities using specific sensors. Thus, a subject could perform a cognitive task while cycling for example, and simultaneously digital biomarkers could be gathered, tracing what happens as it would be in daily activity. Digital biomarkers are objective and quantifiable patient data related to the behavior experienced in the VE (i.e., kinematics) and the physiological states associated with it, such as electroencephalogram (EEG), electrocardiogram (ECG), blood volume pulse (BVP), and respiration signal (RSP), detected by biosensor and medical signals. Through these variables clinicians could derive important information about neurophysiological changes (Hausdorff, 2007; Gates and Valenzuela, 2010), alterations in brain volume in specific areas (Tian et al., 2017), and consequent cognitive decline. In fact, particular patterns of gait kinematics such as slower gait, longer stride time, and more stride-to-stride variability are closely related to cognitive impairment and motor risks, with also a strong association between gait and heart rate dynamics (Hausdorff, 2007). Investigating these aspects in conjunction with cognitive requests may help to determine some clinical crucial aspects like disease severity, medication utility, and objectively document improvements in response to therapeutic interventions, above and beyond what can be gleaned from traditional measures.

## How Could This Integration Take Place?

The massive amount of information extrapolated from a patient during her/his diagnosis and treatment process needs a similarly powerful technology to process it and convert it into an output intelligible for both clinicians and patients. Machine Learning (ML) may be the most appropriate technique for managing a large volume of different, complex, and extensive data. Artificial Intelligence (AI) is a computer science field that performs activities capable of replicating human performance, such as learning to understand complex data, which is a process that requires human intelligence (Bawack et al., 2019; Graham et al., 2019; Wang, 2019). ML algorithms can discover and predict data trends and patterns by building on existing information and highlighting unexpected relationships between variables, without requiring *a priori* hypotheses about their relationships (Graham et al., 2019). Thus, ML is associated with the study and construction of systems that can learn on their own rather than following instructions. This “learning by processing” approach generates increasingly accurate predictive models for diagnosis, individual prognosis, and risk estimation (Vieira et al., 2017; Dwyer et al., 2018; Facal et al., 2019). The use of ML in healthcare has been divided into two categories: supervised (SL) and unsupervised (UL) techniques. To decide which feature best predicts the pre-labeled data, SL uses both pre-labeled data (e.g., cognitive impairment vs. healthy participants) and extra features acquired from clinical or neuroimaging sources (Dwyer et al., 2018; Graham et al., 2020). UL techniques, instead, sets unlabeled and unstructured data, e.g., clinical notes, as a starting point to seek relationships or patterns and to learn general representations that enable the automatic extraction of information when building predictors (Miotto et al., 2016; Dwyer et al., 2018; Graham et al., 2020). The great opportunity offered by AI is to combine a great number of various data, thus cognitive and motor indices may be integrated with digital biomarkers detected in the VE, offering more reliable and predictive neurophysiological results compared to the classic paper and pencil tests. AI could optimize individual treatment strategies by applying ML techniques, helping the transition to personalized, effective, and engaging medicine, built on the individual patient’s needs. In the literature, ML is linked to diagnostic screening tools with subsequent analysis of the progression of the disease (Tack, 2019; Cavedoni et al., 2020; Charvatova et al., 2020). An innovative approach is applying ML to rehabilitation, allowing precise predictions on which motor or cognitive parameters are more predictive for the future maintenance of any improvements obtained. Treatment Effect Prediction (TEP) is significant in disease management because it ensures that patients receive the expected clinical outcomes after undergoing specialized and complex treatments based on their unique clinical condition. However, there are still very few studies that analyze the potential of TEP (Shi et al., 2019; Chu et al., 2020; Wilfling et al., 2020); the latter are in the pharmacological field and use ML to predict possible side effects. Therefore, ML can augment the ability of healthcare providers to improve patient care, deliver accurate diagnoses, optimize treatment plans, inform decisions or allocate resources within health systems: precision medicine is a revolutionary approach already present in the field of pharmacology (Wilfling et al., 2020).

## A Proposed Clinical Application

We suggest that VR would be the best scenario for integrating the two crucial aspects guiding daily life and those important behavioral characteristics and physiological activation. These features may be examined systematically and accurately using ML in conjunction with neuropsychological and clinical data. This comprehensive strategy could allow for the rehabilitation and the monitoring of all factors associated with aging decline. In this section, we will propose an example of this new integrated approach in the rehabilitation field.

The training will be carried out on elderly people with frailty syndrome and cognitive impairment. Participants will be randomly allocated to one of the two conditions: experimental and Treatment as Usual (TAU). Both trainings will be delivered throughout ten one-hour sessions. In TAU condition participants will complete both a paper and pencil neuropsychological rehabilitation and classic motor exercises. The experimental condition consists instead of an active VR dual-task played in the CAVE wearing 3-D glasses at IRCCS Istituto Auxologico Italiano. This immersive technology includes cameras and several head-tracked, as well as a variety of physiological and motion metrics for quantifying embodiment and movements in the VE. The protocol will be drawn based on existing tools (Pedroli et al., 2018, 2019a,b), involving different dual-task exercises: the Positive Bike, Rocks, and the Supermarket.

a. In the Positive Bike exercise, patients will use a stationary bike placed inside the CAVE. They had to maintain a constant cycling speed (motor task) and distinguish target objects from distractors (cognitive task). The exercises parameters (e.g., time between targets presentation, the target to select, bike velocity) will be decided by the therapist in each session.
b. The Rocks is designed to improve balance by immersing patients in a VE that resembles a straight road and requiring them to avoid rocks (motor task). We will add a cognitive part to this exercise: subjects will have to announce their direction while moving (i.e., if rocks move to the right, they’ll say “right,” and if they move to the left, they’ll say “left”).
c. The Supermarket exercise involves executive functions training in a virtual supermarket where patients will use an X-Box controller to move around the store and purchase many things while adhering to strict regulations. There will be ten distinct activities to choose from, each with a different level of difficulty. While shopping, we will add a motor task consisting of a walk-in place with a metronome.

**FIGURE 1:**
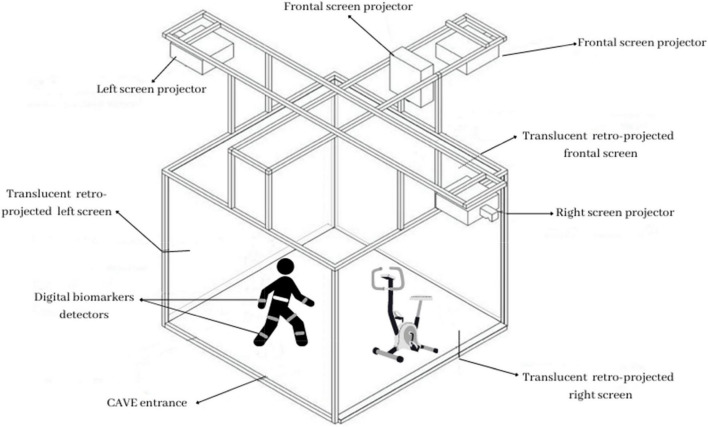
Subject’s digital biomarkers detected within a VR environment (CAVE) while performing DT.

During both training conditions, digital biomarkers will be collected. At the beginning of each session, all participants will wear kinematic motion detectors to detect specific bodily movements, such as velocity and stride variability. They will be attached with an elastic belt and a velcro closure to a participant’s trunk and bilateral thigh and shank. Additionally, they will wear a particular h-Health smartwatch to measure heart rate variability and a chest strip sensor that will collect cardiovascular and respiratory activity. Figure 1 shows a schematic illustration of the technological equipment.

**FIGURE 2:**
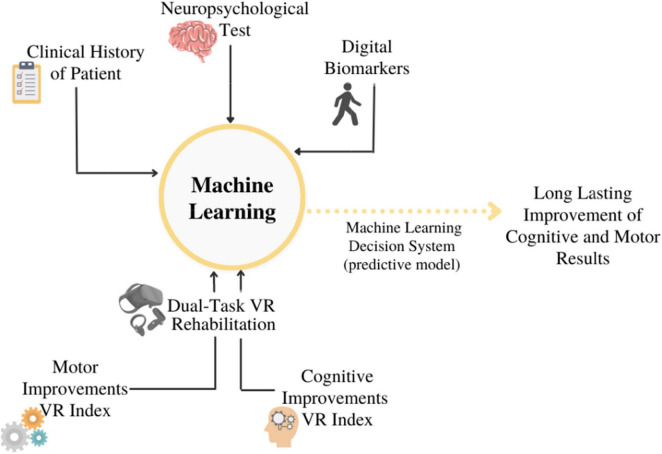
Schematic illustration of the innovative model proposed.

A neuropsychological and motor assessment will be carried out before (T0), immediately after (T1), three (T2), and six (T3) months after the end of the 10 sessions, to evaluate the long-term efficacy of the treatment. To analyze data, we will assume the use of SL techniques: the goal would be to find a link between pre-labeled data and each rehabilitation session’s clinical motor and cognitive findings. (i.e., clinical history of patients, standard neuropsychological battery results, and digital biomarkers). Two different types of ML will be tried out: an ML model built with the personal data set and another ML model built with the total data set. A schematic illustration of this model is depicted in Figure 2. In this case, however, the intent will be to show if there is any predictive long-term maintenance improvement. This would allow researchers to determine whether VR dual-task focuses on maximizing the positive effects of rehabilitation. Indeed, ML is the perfect tool, which allows analyzing the large amount of several data available: a mix of the history of the patient, results of the traditional neuropsychological test, and digital biomarkers. ML will also support the multidimensional and integrated approach aimed to use rehabilitation results and data of patients’ clinical history as the input data to train a strategic dataset that in future could discriminate new cases. Particularly, using different algorithms, (e.g., Logistic Regression, Naive Bayes, Random Forest) ML would be able to track the progress of the rehabilitation of patients, its effects, and effectiveness based on the clinical history, neuropsychological tests, and parameters of digital biomarkers measured.

## Discussion

Aging may be a particular source of vulnerability in frailty individuals and older adults with some cognitive difficulty which may blur the border between normal and pathological aging, increasing the likelihood of chronic disease (Lutz et al., 2008). Thus, early interventions are crucial for preserving cognitive functioning and, as far as possible, slowing the progression of dementia and physical impairment. When the human system is functioning properly, a multi-level integrated control system combines information from the brain with feedback from visual and proprioceptive sensors to produce, control, and modify motor patterns in response to environmental changes (Hausdorff, 2007). In this panorama, the combination of dual-task techniques with a fully immersive technology would presumably help in providing more precise results in terms of assessment. This allows for identifying real lifelike problems, and consequently, treating them with better outcomes compared to a vertical intervention because it implies the joint presence of neuropsychological and motor abilities within a realistic experience. Given the potential of VR, as well as fast feedback and repetitive practice, sophisticated cognitive processes would be facilitated by enjoyment and attractiveness, which might help motivate and engage users (Tuena et al., 2020). Patients would have to employ their attentional, mnemonic, planning, adaptability, and navigational skills to perform the virtual assignments. Thus, it is reasonable to assume that a multisession and multimodal intervention will help with the transfer of these skills to real-life daily tasks. These expected results are consistent with prior research, which has shown that performing two tasks at the same time has greater positive benefits than performing one activity at a time or sequential training (Tait et al., 2017). Further, DT is expected to increase executive functions, locomotion, and daily complicated activities in general (Mirelman et al., 2011; Wang et al., 2015; Liao et al., 2019). On these bases, the current perspective seeks to propose an innovative approach that incorporates different components while utilizing new technical devices considering both motor and cognitive baseline and improvements. Indeed, VR potential lies in duplicating lifelike behaviors (such as cycling in a park and shopping) in an ecological and controlled setting. Defining problems in aging that are as similar as in daily life allows clinicians to provide individual training that could track the specific needs of the patient that could be treated in an ecological way instead of standard interventions generally proposed in clinical practice. However, one of the problems of rehabilitation is maintaining improvements; hence, it is necessary to assess whether and how improvements will be maintained, taking into account the diversity of the results (Pashler et al., 2001; Verghese et al., 2007; Schaefer et al., 2015). To address this issue, the present perspective proposes a predictive model for detecting specific patterns of cognitive and motor impairment in aging populations and predicting outcomes. Further, digital biomarkers could also facilitate an advanced outcomes prediction and personalization of the therapeutic approach, guaranteeing customization and effective indicators of biologic processes or responses to therapeutic intervention. Indeed, sensor-based gait variability parameters were identified as clinically most relevant digital biomarkers for gait impairment (Hausdorff, 2007; Cavedoni et al., 2020; Gaßner et al., 2020) and a strong association between gait and heart rate dynamics have been identified (Hausdorff, 2007). Investigating these aspects in conjunction with cognitive requests may help to determine some clinical crucial aspects such as disease severity, medication utility, and to objectively document improvements in response to therapeutic interventions, as well as specific parameters like the difficulty and sessions, useful for effective personalized training. AI learning by processing may enable for selection of the most predictive variables, which will subsequently be empirically verified *via* follow-up; it will be also improved, based on the historical data of patients, results of neuropsychological traditional tests, and digital biomarkers measured. Measures from the initial evaluation can be compared to ML clinical data and cognitive-motor characteristics to see if and how their baseline condition influenced and predicted the outcome of rehabilitation. These integrations of different data methodologies and techniques may improve the treatment’s reliability in terms of better prognostic indexes and individualized training. Clinicians might create efficient and individualized training: they could choose the best therapy in terms of intensity or number of sessions, for example, they could collect information easily about the possible future directions of the patients in terms of treatment and cognitive/motor development and saving patients’ time to choose appropriate intervention in the assessment phase, by providing training quickly and efficiently. This approach crosses standardized therapy provided usually in a pre-structured way. Specific and well-timed interventions are essential to retain cognitive functioning and slow the progression of dementia and physical deterioration. For instance, early detection of gait deficits allows the early revelation not only of motor risk but also cognitive deficit, with the possibility to implement timely intervention (Lunardini et al., 2021). Further, in some cases, it could be more effective to use a vertical therapy than a traditional dual-task or a VR dual-task, allowing for long-term improvement in rehabilitative outcomes.

The integrative model of rehabilitation proposed, however, could be useful also in the assessment since data may provide the starting point for future intervention and to evaluate the person’s progression, considering assessment and training as a continuum in the patient healthcare. This approach demonstrates how research and clinical practice are intertwined: research supplies the instruments, while practitioners provide critical data to confirm research creating a closed-loop system that allows researchers and clinicians to effectively interact with each other.

In summary, while much of the literature has focused on different methodologies addressed to the needs of the aging population, little was done considering the individual not as a circumscribed problem, but as a whole single person composed of mind and body at the same time. Furthermore, no one has looked at it and how some predicted characteristics are linked to the patient’s fragility at the outset, opening up new avenues. In this paper, cognition meets gait in a dual-task approach that considers the individual in all her/his whole aspects. On one hand, VR provides the physical space in which all data can be collected with a high degree of ecological validity. On the other hand, ML provides the best procedures aimed to improve quality of life by lowering healthcare costs and hospitalization rates, hence expanding primary, and secondary prevention options, in the perspective of personalized medicine.

## Data Availability Statement

The original contributions presented in this study are included in the article/supplementary material, further inquiries can be directed to the corresponding author.

## Author Contributions

FBr, EP, and PC developed the new model integrating virtual reality and machine learning. FBo supervised the sections of machine learning. VM supervised the section regarding virtual reality. FBr wrote the manuscript under the final supervision of EP and PC. GR and MS-B provided the required revisions. All authors have approved the final version of the manuscript.

## Conflict of Interest

The authors declare that the research was conducted in the absence of any commercial or financial relationships that could be construed as a potential conflict of interest.

## Publisher’s Note

All claims expressed in this article are solely those of the authors and do not necessarily represent those of their affiliated organizations, or those of the publisher, the editors and the reviewers. Any product that may be evaluated in this article, or claim that may be made by its manufacturer, is not guaranteed or endorsed by the publisher.
